# Measuring Bismuth Oxide Particle Size and Morphology in Film-Coated Tablets

**DOI:** 10.3390/molecules27082602

**Published:** 2022-04-18

**Authors:** Stefani Fertaki, Georgios Bagourakis, Malvina Orkoula, Christos Kontoyannis

**Affiliations:** 1 Department of Pharmacy, University of Patras, GR-26504 Rio Achaias, Greece; sfertaki@iceht.forth.gr (S.F.); malbie@upatras.gr (M.O.); 2 Institute of Chemical Engineering Sciences, Foundation of Research and Technology-Hellas (ICE-HT/FORTH), GR-26504 Platani Achaias, Greece; 3 ELPEN S.A. Pharmaceutical Industry, 5 Marathonos Ave., GR-19009 Pikermi Athens, Greece; gbagourakis@elpen.gr

**Keywords:** bismuth oxide, API particle size, API morphology, film-coated tablets, Raman spectroscopy, ImageJ, tablet disintegration

## Abstract

The assessment of active pharmaceutical ingredient (API) particle size and morphology is of great importance for the pharmaceutical industry since it is expected to significantly affect physicochemical properties. However, very few methods are published for the determination of API morphology and particle size of film-coated (FC) tablets. In the current study we provide a methodology for the measurement of API particle size and morphology which could be applied in several final products. Bismuth Oxide 120 mg FC Tabs were used for our method development, which contain bismuth oxide (as tripotassium dicitratobismuthate (bismuth subcitrate)) as the active substance. The sample preparation consists of partial excipient dissolution in different solvents. Following this procedure, the API particles were successfully extracted from the granules. Particle size and morphology identification in Bismuth Oxide 120 mg FC Tabs was conducted using micro-Raman mapping spectroscopy and ImageJ software. The proposed methodology was repeated for the raw API material and against a reference listed drug (RLD) for comparative purposes. The API particle size was found to have decreased compared to the raw API, while the API morphology was also affected from the formulation manufacturing process. Comparison with the RLD product also revealed differences, mainly in the API particle size and secondarily in the crystal morphology.

## 1. Introduction

Particle size of pharmaceutical powders can have a significant effect on the safety and efficacy of the drug product. The particle size distribution (PSD) and morphology of APIs have a profound impact on the drug product performance including dissolution, bioavailability, and formulation stability [1,2,3,4]. Additionally, the PSD of the API powders may also influence the drug flowability, blend uniformity, compactibility, or other characteristics at almost every step of the manufacturing process, including mixing, granulation, and compression [4,5,6]. Therefore, the particle sizes and morphology of pharmaceutical powders should be evaluated at different pharmaceutical development phases and primarily in the final product.

A number of methods are available for determining particle size of which the most common include sieve analysis, laser diffraction, dynamic light scattering, and direct imaging techniques [7]. The most common method for particle size distribution is laser diffraction measurement [8]. However, such techniques are used for APIs’ PSD prior to any manufacturing processes and tableting. Very few studies have been published regarding the measurement of API particle size and morphology in pharmaceutical final products. Michal Šimek et al. have used hot-stage microscopy in order to melt the excipients present in tablets containing tadalafil or meloxicam API. After successful disintegration of the tablets using 0.5 mL of water, the remaining powder was placed under the microscope and onto the hot-stage where it was heated until complete melt of the excipients. The API particles were then observed and measured using image analysis [9]. This procedure exhibits some limitations since the API melting point should be higher than the rest of the excipients. Usually though, the temperature of API melt is not very high (~200 °C) in contrast to the excipients. Atsushi Kuriyama and Yukihiro Ozaki managed to identify the PSD of Ebastine API in Ebastel tablets using micro-Raman chemical imaging and polystyrene microsphere size standards as reference. They have applied a specific binarization threshold value compared to the reference tablets containing microspheres in order to calculate the PSD of the API particles. However, this approach can work only if API particles are well dispersed into the formulations and the histogram indicates no API-specific distribution, and all pixels are a mixture of components with the API maximum intensity prior to formulation not being retained. Additionally, the morphology of the API crystals cannot be clearly observed [10].

A few additional publications used Raman imaging for the determination of API particle size distribution in pharmaceutical formulations [11,12,13]. However, this technique by itself could be more useful for the identification of the ‘domain’ particle size since the discrimination of frequently observed agglomerated particles cannot be easily performed.

In the present work, the bismuth oxide API particle size and morphology in Bismuth Oxide 120 mg FC Tabs were studied. This medicine is used for the treatment of peptic ulcer and gastroesophageal reflux disease and also shows antibacterial activity against Helicobacter pylori [14]. Several solvents were used to dissolve the main excipients and release the API from the granules. The sample was spread in a highly reflective sample carrier at 40 °C in order to dry while micro-Raman mapping was performed to identify all the remaining particles. The identified API particles were then measured with ImageJ software. The particles’ morphology was also studied. Comparison with an RLD product was also performed. The effect of the manufacturing process and sample preparation in the API particle size and morphology was analyzed as reference.

## 2. Results

### 2.1. Sample Preparation for Bismuth Oxide 120 mg Film-Coated (FC) Tabs Disintegration and API De-Aggregation

The tablet core of Bismuth Oxide 120 mg FC Tabs consists of API, maize starch, povidone K29/32, polacrilin potassium, macrogol 6000, and magnesium stearate (Table 1). The API content is approximately 30% *w*/*w*.

The tablet preparation was performed using dry granulation. The API along with maize starch, macrogol 6000, and PVP were premixed and granulated using a roller compactor. The produced compacts were sized and mixed with extragranular PEG 6000, polacrilin potassium, and magnesium stearate in order to prepare the lubricated blend. Finally, the blend was compressed to tablets before the coating step. 

**Figure 1:**
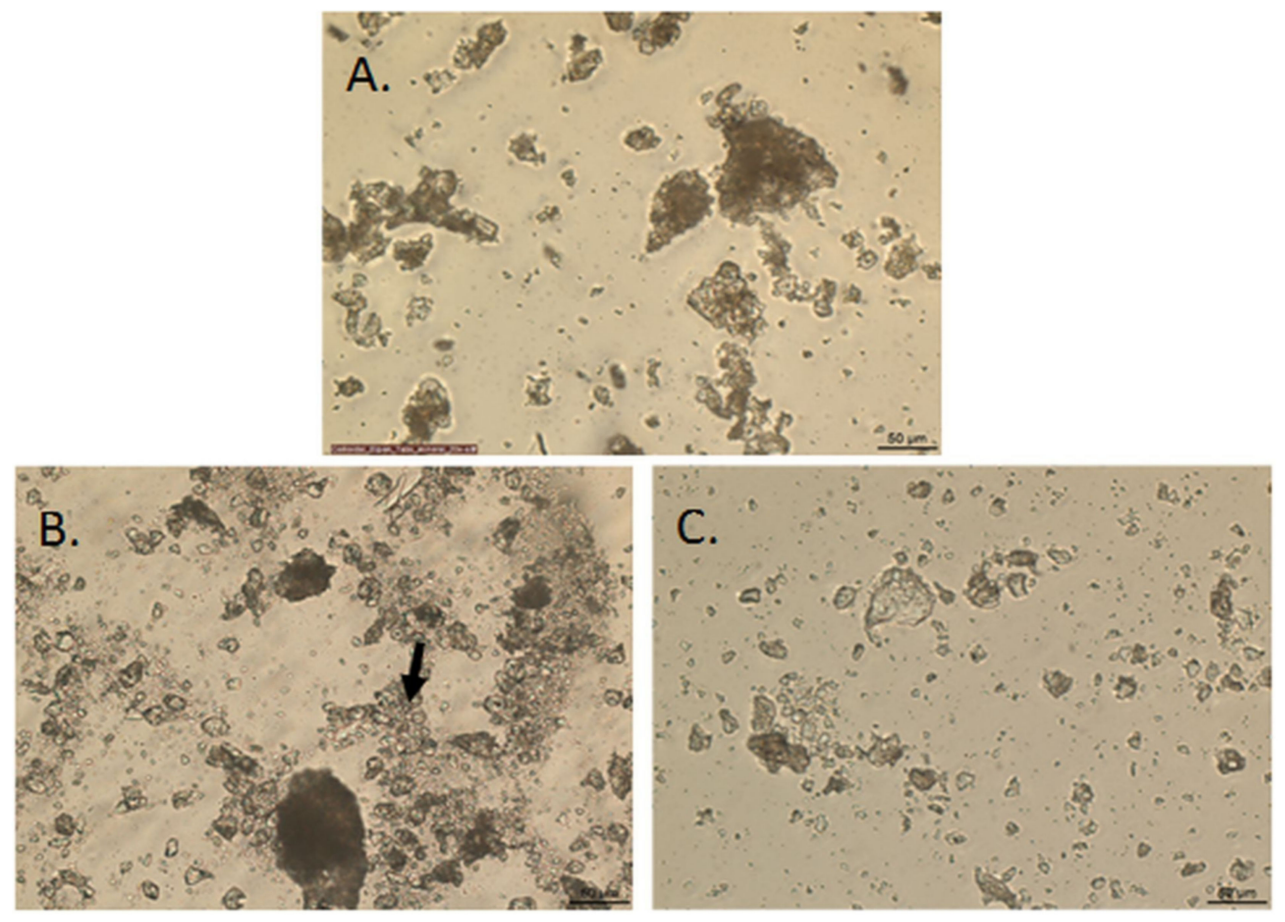
Optic microphotographs of Bismuth Oxide 120 mg FC Tabs as received after film removal (**A**), after treatment with acetonitrile (**B**), and after treatment with acetonitrile and subsequent treatment with heated DMSO (**C**) using 20× magnification objective lens.

In order to eliminate the excipients’ presence, the film coating was initially removed with a scalpel. The main difficulty of the current task was the successful API extraction from the granules without damaging the API crystals. Literature revealed several solvents that could potentially dissolve some of the excipients, whereas the API remained insoluble. Using both literature and a trial-and-error process, it was observed that acetonitrile managed to dissolve povidone K30 and macrogol 6000. The images obtained from the suspended Bismuth Oxide 120 mg FC Tabs in acetonitrile revealed some remaining agglomerates and possible granule presence though (Figure 1B). Additionally, the dispersion was dominated with maize starch presence (spherical particles noted with arrow in Figure 1B). 

Maize starch was therefore considered as the next excipient target since it is a granulation binder. Heated dimethylsulfoxide (DMSO) can dissolve maize starch and form a viscous colloidal solution. The latter was verified when pure maize starch was diluted in DMSO at 80 °C while the API was once again unaffected. As seen in Figure 1C, the agglomerates were almost absent. Polacrilin potassium and Mg stearate could not be dissolved with any of the known solvents without affecting the API. 

**Scheme 1:**
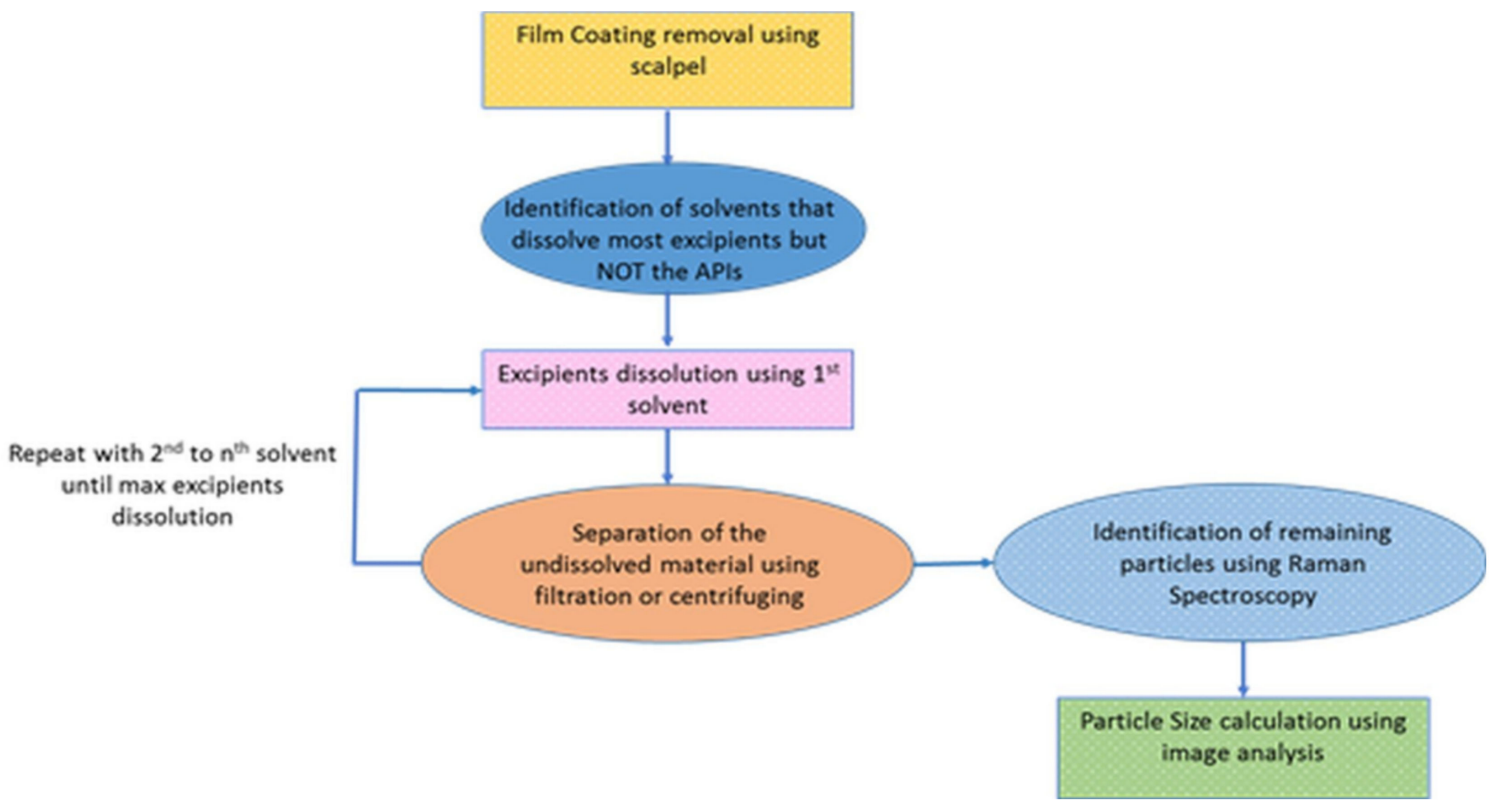
Flow chart describing the methodology used for API particle size and morphology identification.

Considering the previous information, the following procedure was performed as the most adequate in terms of sample preparation for tablet disintegration and consequent API extraction from the granules (also presented in Scheme 1 as flow chart).

After film removal, the tablet was cut into eight smaller pieces using a scalpel in order to avoid direct crushing and consequently damaging the API particles. Then, approximately 100 mg of the sample (four pieces) was dispersed in 6 mL acetonitrile in falcon tubes. The dispersion was vortexed for 5 min at 2500 rpm until complete powdering of the tablet pieces. The sample dispersion was then filtered and left to dry at 25 °C for 1 h. The remaining powder was then dispersed in heated DMSO (80 °C) for 2 h in a closed vial. The dispersion was then centrifuged in order to remove the DSMO solution. The remaining powder was re-dispersed in fresh DMSO in order to obtain a homogeneously dispersed mixture of the sample and to assist in obtaining clearer images. A few drops of the dispersion were added on a highly reflective gold glass slide which was placed on a hot-stage at 60 °C in order to dry the particles for the following Raman study, since the presence of liquid can affect the Raman signal and result in unsuccessful discrimination of the particles.

### 2.2. Particle Identification Using Micro-Raman Mapping Spectroscopy

The obtained particles from the previously stated procedure were further investigated using micro-Raman spectroscopy in order to be identified.

**Figure 2:**
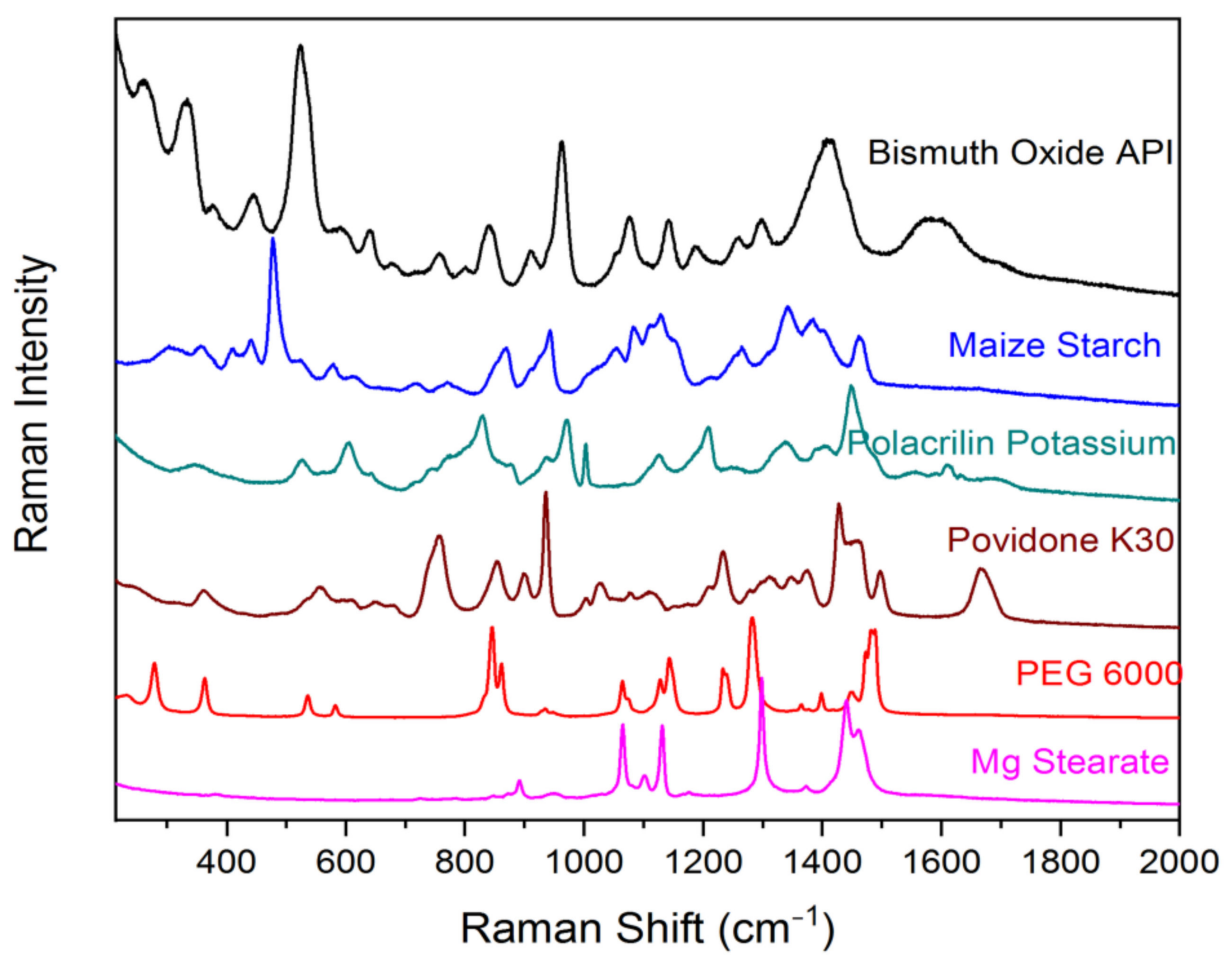
Raman spectra of bismuth oxide API, maize starch, polacrilin potassium, povidone K30, PEG 6000, and magnesium stearate.

Raman spectra of pure samples of bismuth oxide API and the excipients are shown in Figure 2. Their spectral differences are distinct, therefore, Raman spectra can be used for discrimination among the remaining particles.

**Figure 3:**
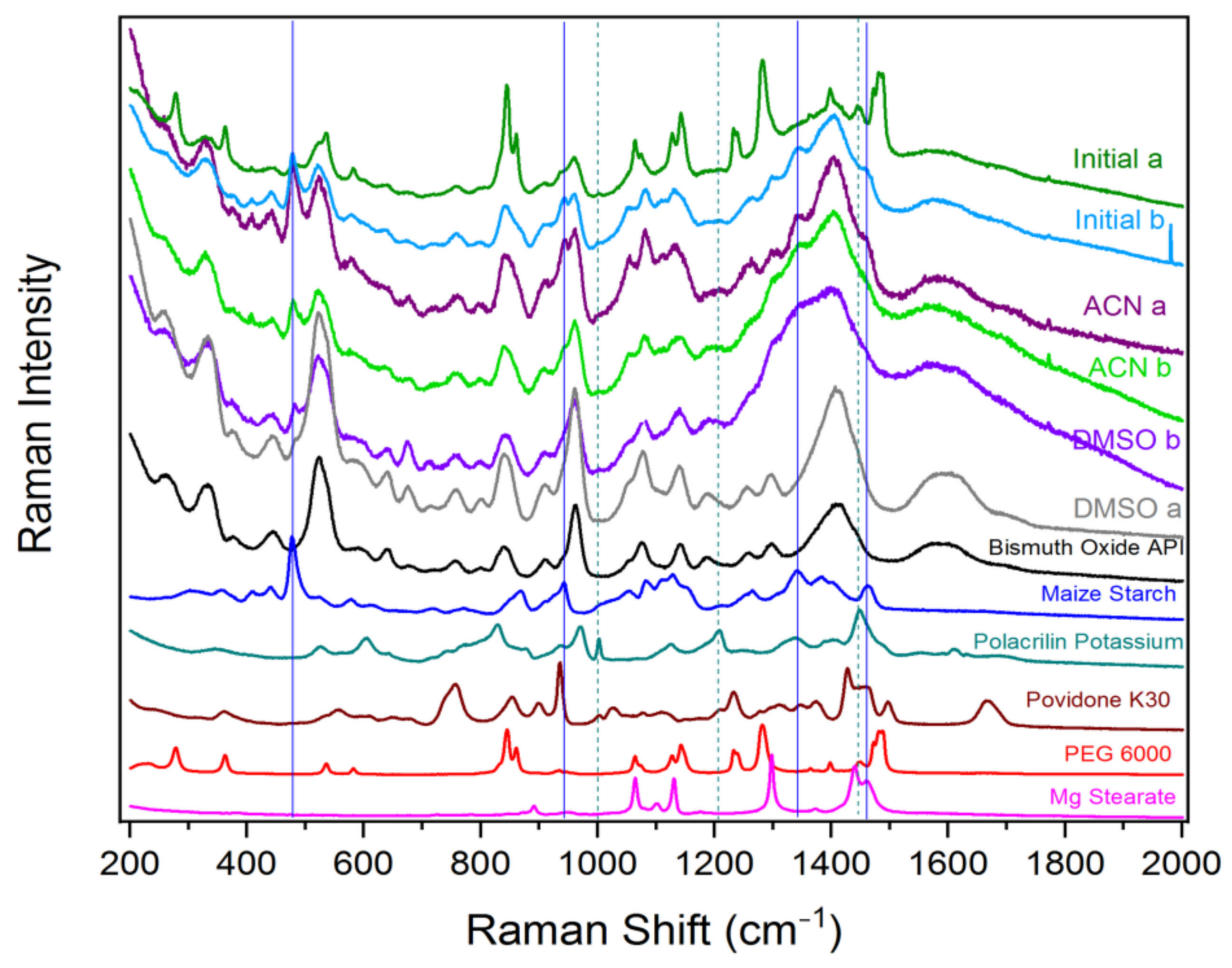
Raman spectra of bismuth oxide API, excipients, initial tablet, tablet dispersed in acetonitrile, and tablet further dispersed and centrifuged in heated DMSO.

In Figure 3, the spectra of the initial tablet before any treatment were compared with the respective spectra obtained from the tablets dispersed in acetonitrile (ACN) and after DMSO dispersion in order to verify the extinction of the dissolved excipients at each step. As expected, the Raman spectra of the initial tablets were combined by bismuth oxide API, maize starch (blue lines in Figure 3), polacrilin (cyan dotted lines in Figure 3), and PEG 6000. After dispersion in acetonitrile, the resulting Raman spectra were dominated by API, some maize starch, and some polacrilin. Finally, after DMSO dispersion, the Raman spectra of the tablets were dominated by API. In some parts, some polacrilin and some remaining maize starch peaks were noticed. It was thus assumed that, indeed, the respective sample preparation could result in partial excipient dissolution and API extraction from the granules.

**Figure 4:**
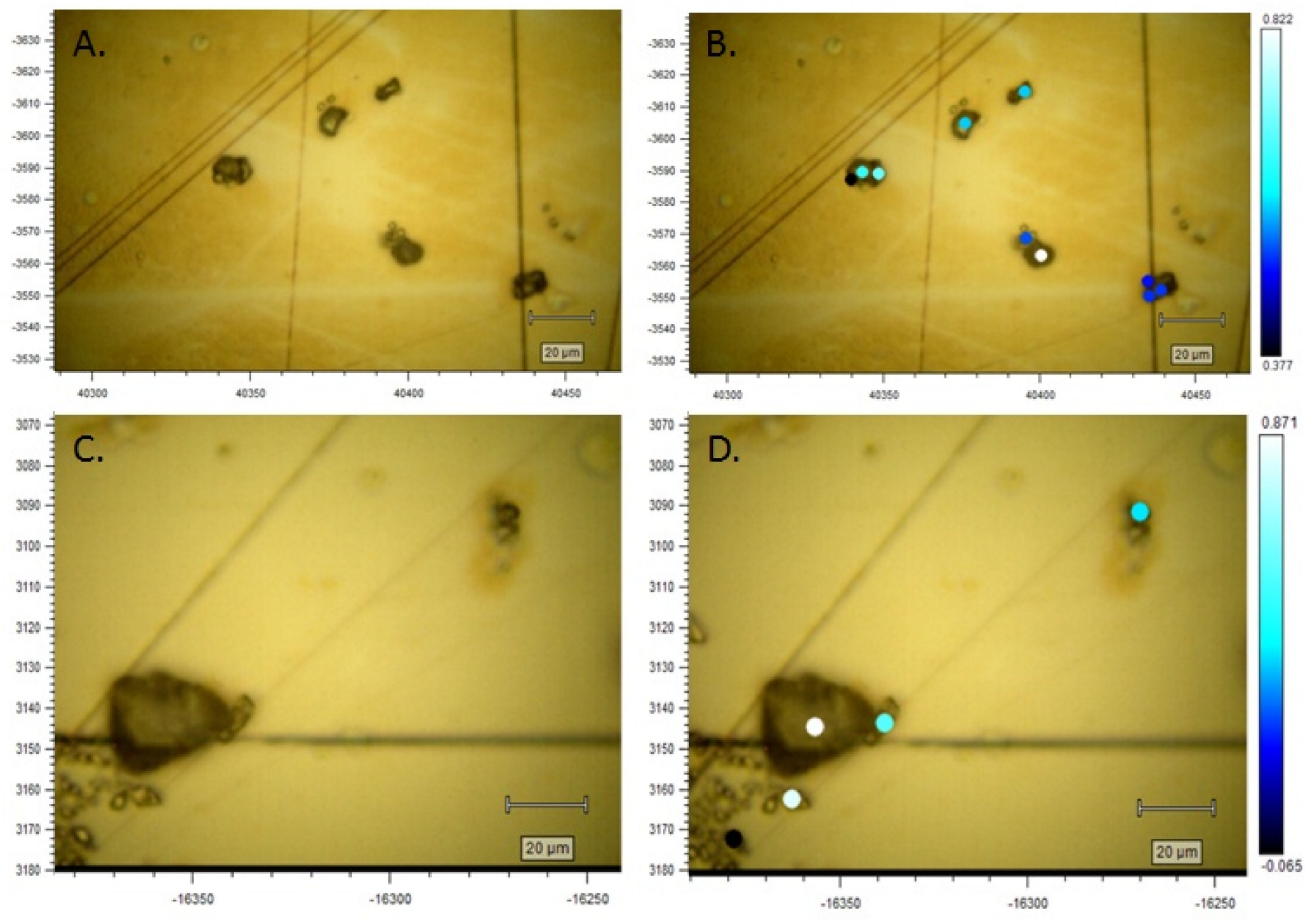
Images of the first and second random mapped area of Bismuth Oxide 120 mg FC Tabs after dispersion in original mode (**A**,**C**) and after processing with direct classical least squares (DCLS) method (**B**,**D**) using 50× magnification objective lens.

A point-to-point mapping procedure was performed in order to scan a total number of at least 400 particles. The data from each pixel were analyzed using direct classical least squares (DCLS) method using API as the reference spectrum. Visualized score maps were thus generated with white and light blue pixels indicating high threshold values (higher than 80% score) corresponding to the API, and the blue pixels (lower than 80% score) indicating low values corresponding to other components (Figure 4). The Raman spectra from all pixels were compared against the API and the excipients (Figure 4). The analysis was performed in duplicate for statistical purposes.

**Figure 5:**
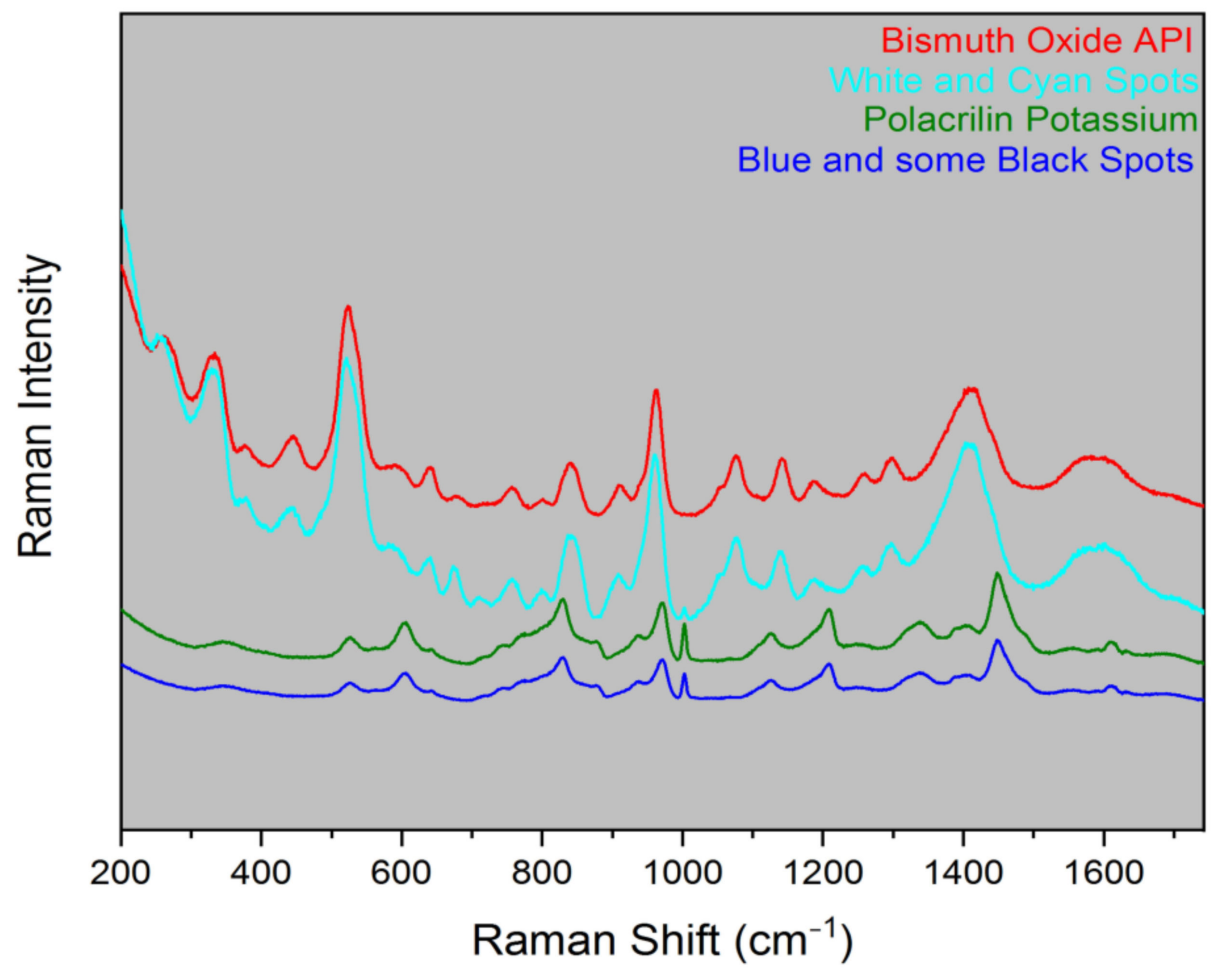
Typical Raman spectra of the particles marked with white and cyan spots (cyan line), blue spots (blue line), and black spots of Figure 4 (black line), colloidal bismuth tripotassium dicitrate (red line), polacrilin potassium (green line), and DMSO (grey line).

From Figure 5, it is apparent that all white and light blue spots correspond to API particles whereas most of the other spots (Figure 4) are attributed to polacrilin potassium particles. Magnesium stearate was not observed, probably because it exhibits a small particle size while at the same time it is present at a very low content in the final products (Table 1). The API particles were predominant in all scanned areas. Particles of irregular shape were observed exhibiting a different morphology from the API particles initially added in the formulation (see Section 2.4).

### 2.3. API Particle Size Determination Using ImageJ

**Figure 6:**
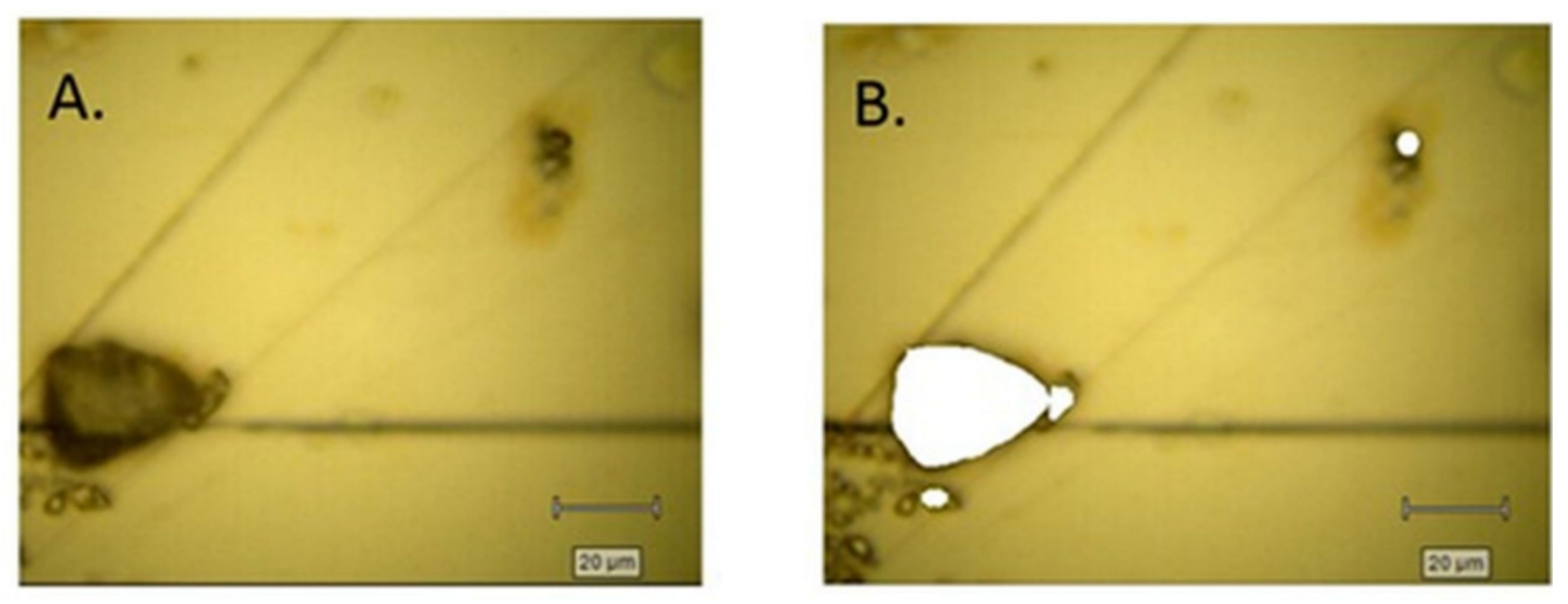
Images of one random mapped area of Bismuth Oxide 120 mg FC Tabs after dispersion in original mode (**A**) and after image analysis method (**B**) using 50× magnification objective lens.

The particle size of the already identified API particles with Raman spectroscopy was measured using ImageJ software. The area of each individual API particle was calculated manually using free-hand selection and drawing the particle’s perimeter (Figure 6). The software automatically calculated the area of each particle and particle’s diameter. The procedure was repeated using all areas previously scanned with Raman spectroscopy until at least 400 API particles were measured in duplicate. The D[0.1], D[0.5], and D[0.9] values of the sample were finally determined using the appropriate mathematic equations (Table 2).

### 2.4. Effect of Sample Preparation in Bismuth Oxide API

**Figure 7:**
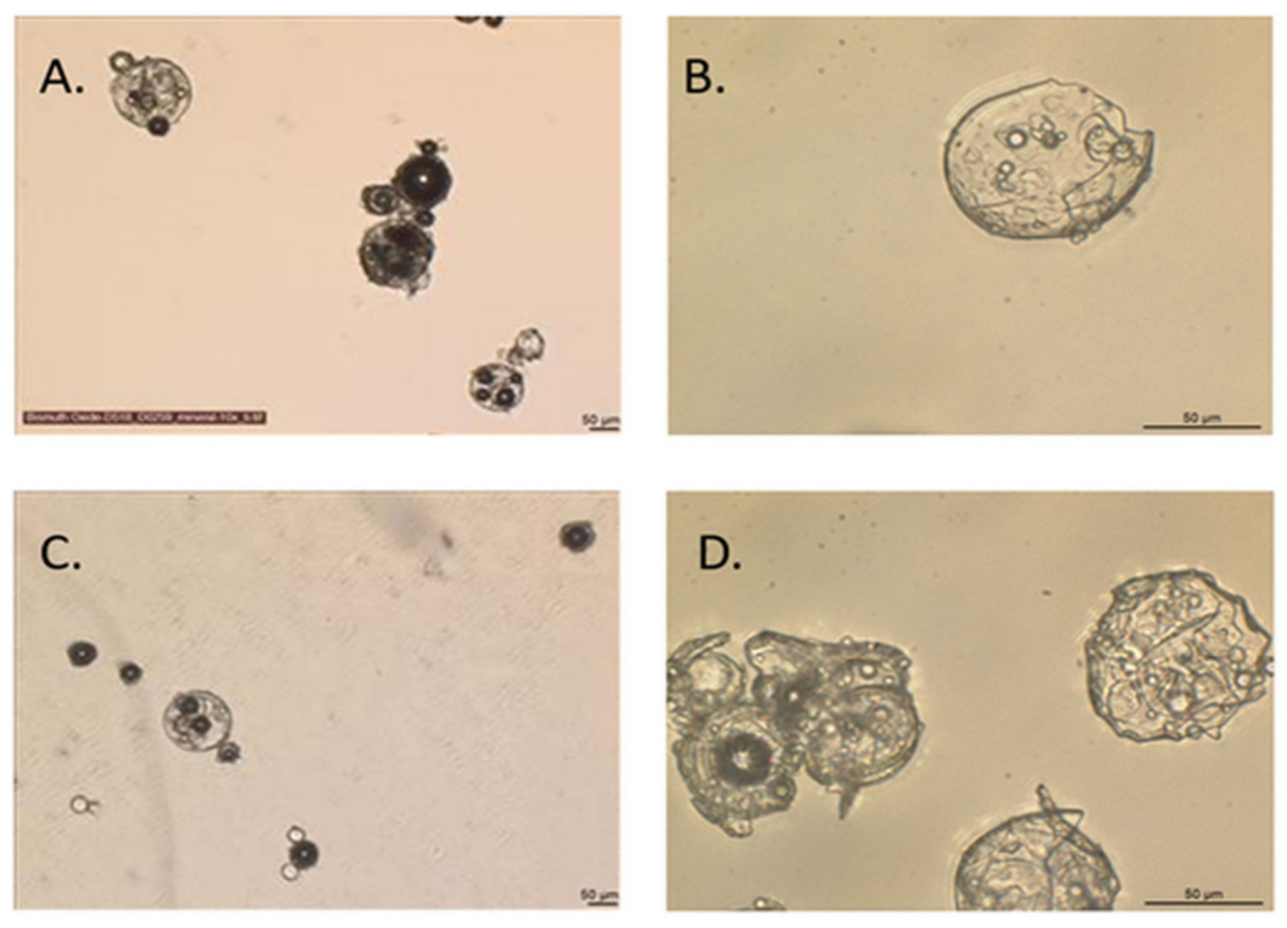
Optic microphotographs of original bismuth oxide API using 10× magnification objective lens (**A**) and 40× magnification objective lens (**B**), and after treatment with acetonitrile and subsequently with DMSO particles using 10× magnification objective lens (**C**) and 40× magnification objective lens (**D**).

The effect of the current manufacturing process in the morphology and particle size of the pure API was also studied. Initially, the morphology of the API was studied from the original powder and after the application of the sample preparation method described in Section 2.1 using optical microscopy and LAS image analysis (Figure 7) on the pure API particles. The particles appeared almost identical and mostly spherical in both cases indicating that the suggested sample preparation scheme does not affect the API’s crystal morphology.

In order to verify that the particle size of the API was not affected from the sample preparation process, the PSD of the API was measured before and after the application of the proposed method using laser diffraction technique. From Table 3 and according to ICH guidelines [15] it was concluded that the PSD of the API was not significantly affected by the respective sample preparation method.

### 2.5. Effect of Final Formulation Manufacturing Process on Bismuth Oxide API Particle Size

**Figure 8:**
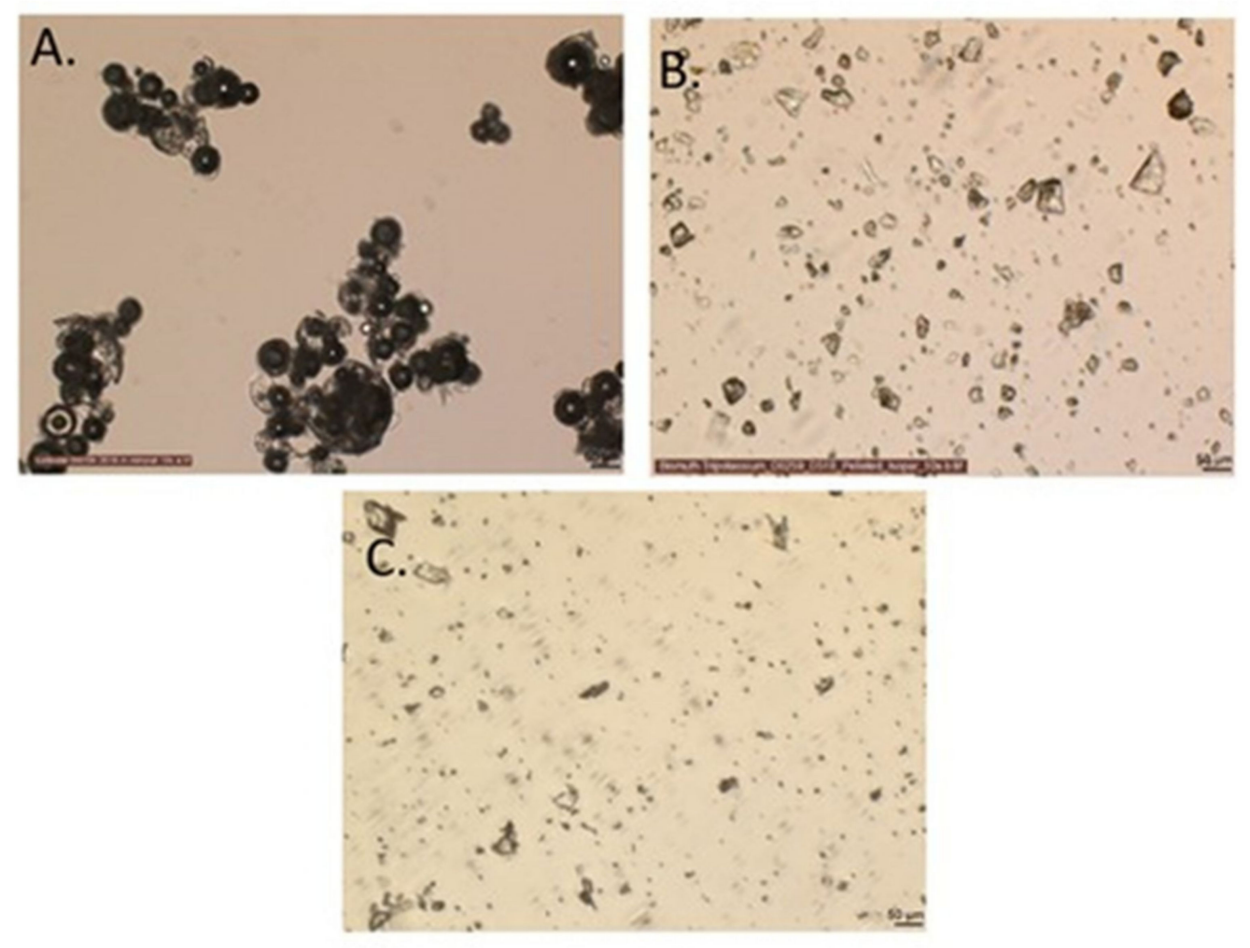
Optic microphotographs of original bismuth oxide API (**A**), pressed at 1 ton API (**B**) and pressed at 10 tons API (**C**) using 10× magnification objectives.

A significant difference was noticed in the API particle size calculated in the final formulation compared to the initial as-received API. According to the study performed in Section 2.4, the proposed sample treatment did not affect either the morphology or the particle size of the API. It was therefore suspected that the compression applied during the manufacturing process of Bismuth Oxide 120 mg FC Tabs could damage the API particles and consequently yield decreased particle size values. For this purpose, the morphology and particle size of the API were analyzed before and after pressure application at 1 ton and 10 tons. From Figure 8 and Table 4 it was verified that bismuth oxide API is significantly sensitive under any amount pressure, justifying the decreased API particle size observed in the final products.

### 2.6. Comparison against an RLD Product

The proposed sample preparation method was repeated for Ulcamed ^®^ 120 mg Fc Tabs (reference listed drug product) in order to identify any possible differences that could result in potential failure of the in vitro equivalence study. The API particle size of the RLD product was found increased in regards to D[0.1] and D[0.1] values according to ICH guidelines Q2R1 compared to the respective Bismuth Oxide 120 mg FC Tabs value (Table 5). Additionally, Ulcamed ^®^ 120 mg tablet disintegration was performed faster than the respective test product. Both these findings indicate possible lower pressure application in the RLD product.

## 3. Discussion

Identification of particle size and morphology of the components present in pharmaceutical formulations is of crucial importance since it is expected to significantly affect the API dissolution rate, bioequivalence, etc. The methodology and sample preparation developed in this study have managed to successfully calculate bismuth oxide API particle size while simultaneous identification of the crystal morphology of the API in prepared Bismuth Oxide 120 mg FC Tabs was also performed.

The experimental procedure was based on the increased API content by dissolving most of the formulation excipients. Attention should be paid to solvent selection because the API must be insoluble in the chosen solvent. Careful transformation from a tablet to powder was also performed, along with proper dispersion of the remaining component particles. The use of a highly reflective sample carrier (gold-plated) favored the API particles identification of the dried suspension using point-to-point mapping Raman spectroscopy. Particle size calculation was finally achieved using image analysis software. Performance of the same procedure on pure API particles revealed that the analysis is harmless for the API particles and therefore appropriate to be used for bismuth oxide pharmaceutical formulations.

Significant changes were noticed in the API particles of the final product compared with the original API particles in terms of size and morphology. Tablet compression seemed responsible for the decreased API particle size and the different crystal shape.

Difference in the API particle size of an RLD product was also confirmed.

The respective method development and sample preparation could be used in the pharmaceutical industry as a routine method for API particle size and morphology determination in film-coated tablets and for other formulations.

## 4. Materials and Methods

### 4.1. Reagents

Τhe commercial film-coated tablets (Ulcamed^®^ 120 mg Filmtabletten), bismuth tripotassium dicitrate, the corresponding placebo, and all the excipients were kindly provided by ELPEN SA (Pikermi, Attica, Greece). The excipients of Ulcamed^®^ 120 mg were similar to our prepared tablet [14].

### 4.2. Preparation of Bismuth Oxide 120 mg Film-Coated Tablets

The process used for the production of Bismuth Oxide 120 mg FC Tabs by ELPEN SA was conventional and adopted standard manufacturing techniques and equipment. Bin mixer (150 L) and roller compactor (Alexanderwerk) were used for the granulation step. Granules compressed using a KILIAN E150 rotary tablet press machine with 10 mm concave punches resulted in tablets with resistance to crushing of approximately 120 N (testing equipment: hardness tester ERWEKA TBH220TD), which were finally coated using Chamunda Pealcoat Ci 900 coater system with a perforated pan.

### 4.3. Dispersions

For the disintegration of a tablet with 120 mg API strength, a dispersion in acetonitrile (HPLC grade) was prepared by dispersing 100 mg of the tablet in 6 mL of acetonitrile. The sample was then vortexed at 2500 rpm using an IKA MS2 Minishaker. Filtration through 0.22 μm GSWP nitrocellulose membrane filters (Merck Millipore Ltd., Cork, Ireland) and a vacuum pump (KNF Neuberger Inc. Laboport, Trenton, NJ, USA) followed. A second dispersion of the remaining undissolved solid in DMSO (80 °C) was prepared by dispersing the undissolved solid in 5 mL DMSO followed by vigorous swirling. The sample was placed in an ultrasonic cleaner (Elmasonic P, P30H, Singen (Hohentwiel), Germany) heated at 80 °C for 2 h.

### 4.4. Raman Spectroscopy

A Raman spectrometer (InVia Reflex Raman spectrometers, Renishaw, UK) equipped with an optical microscope (Research Grade, Leica DMLM microscope) and a laser with a 785 nm excitation line was used. The laser line was focused through a 50× objective lens on the sample. The system was equipped with a CCD detector (Peltier cooled, near-infrared enhanced). The power of the incident laser was 250 mW. The typical spectral resolution was 2cm^−1^. Windows-based software was used (WiRE^©^ 4.2) to obtain the spectra. Instrument response (laser power and the wavenumber) was checked by recording the spectrum of Si.

For Raman spectra collection, the samples were placed on a highly reflective sample carrier (EMF Corporation, Angola, IN, USA). Quantities of a few milligrams for the solids were used. The recorded spectra were the sum of 3 scans of 10 s exposure time and the acquired region was 250–2000 cm^−1^.

For Raman spectra collection of the solutions, 3 drops of the sample were placed on a highly reflective sample carrier (EMF Corporation, Angola, IN, USA) and dried for 3 h in a Leica TPX-Type D hot-stage. A 350 × 500μm area was captured each time and each particles’ spectrum was acquired using a point-to-point mapping until at least 600 particles were measured. The recorded spectra were the sum of 2 scans of 5 s exposure time and the acquired region was 1240–1700 cm^−1^.

### 4.5. Particle Size Distribution (PSD)

Laser analyzer Mastersizer 3000 (Malvern Instruments, Malvern, UK) with Hydro SV adapter was used to determine the PSD of the samples. The measurement range of the apparatus is 0.02–200 µm. Red light of 633 nm wavelength was the source. The stirrer speed was set at 1800 rpm, obscuration range was set at 10–30%, and the particle size was calculated on a volume basis using the Mie theory. Isopar G was used as the dispersant.

### 4.6. Optical Microscopy

An optical microscope (Leica DM 2500M, Leica Microsystems LtD., Heerbrugg, Switzerland) equipped with a video camera (Leica DFC420 C, Leica Microsystems LtD., Heerbrugg, Switzerland) was used to obtain pictures of the API crystals in transmittance mode and brightfield illumination, using the 10× Leica objective lenses; for solutions, the 20× Leica objective lenses were used.

Approximately 3 mg of the powder samples was dispersed in 3 mL of mineral oil (ACRŌS ORGANICS, Geel, Belgium) and the dispersions were analyzed as prepared. One or two drops of the aforementioned samples were then placed on a 76 mm × 26 mm × 1 mm microscope slide (Paul Marienfeld GmbH and Co. KG, Lauda-Königshofen, Germany), covered with a 22 mm × 22 mm cover slip (Paul Marienfeld GmbH and Co. KG, Lauda-Königshofen, Germany). Windows-based software (LAS^©^ V4.11) was used for image acquisition and analysis.

### 4.7. ImageJ

ImageJ software was used for the calculation of API particle size. The Raman obtained images were filtered using enhance local contrast. The area of each particle was automatically calculated after manually drawing each particle perimeter using free-hand selection. The diameter of each particle was acquired from the each measured particle area.

### 4.8. Centrifuge

The samples were placed in a 10 mL falcon tube and were centrifuged at 8000 rpm and 25 °C for 23 min using a refrigerated centrifuge (Heraeus Biofuge Stratos, Kendro, Osterode, Germany). The supernatant, which was approximately 4 mL, was removed and the precipitate was collected.

## Figures and Tables

**Table 1:** Ingredients and content of excipients in placebo used for Bismuth Oxide 120 mg FC Tabs as received from ELPEN SA.

Sample Information	Content (%)	Function	PSD (D[4,3] µm)
Maize starch	58.00	Diluent/disintegrant	-
Macrogol 6000	5.25	plasticizer/lubricant	-
Povidone K-29/39	14.00	Binder	-
Polacrilin potassium	19.25	disintegrant	66.6
Magnesium stearate	3.50	lubricant	10.1

**Table 2:** Average cumulative PSD values of bismuth oxide API particles in 120 mg FC Tabs after disintegration of the tablet.

D[0.1]	D[0.5]	D[0.9]
14.35 ± 0.07 µm	27.00 ± 1.41 µm	43.25 ± 0.35 µm

**Table 3:** PSD values of bismuth oxide API before and after being processed according to sample treatment.

Sample Information	D[0.1]	D[0.5]	D[0.9]
Original bismuth oxide API	32.8 ± 5.1 µm	70.3 ± 7.5 µm	123.0 ± 13.5 µm
API particles treated with acetonitrile and subsequently with DMSO	30.6 ± 3.7 µm	66.3 ± 6.2 µm	121.0 ± 14.1 µm
RSD%	6.71	5.69	1.62

**Table 4:** PSD values (with laser diffraction) of bismuth oxide API before and after pressure at 10 tons.

Sample Information	D[0.1]	D[0.5]	D[0.9]
Original bismuth oxide API	35.8 ± 5.1 µm	71.3 ± 7.5 µm	125.0 ± 13.5 µm
Pressed bismuth oxide API	4.66 ± 1.8 µm	27.7 ± 2.5 µm	54.3 ± 4.8 µm

**Table 5:** Average PSD values of bismuth oxide API using micro-Raman spectroscopy in Ulcamed ^®^ 120 mg FC Tabs against tested Bismuth Oxide 120 mg FC Tabs.

Sample Information	D[0.1]	D[0.5]	D[0.9]
Ulcamed ^®^ 120 mg FC Tabs	14.30 ± 1.41 µm	33.15 ± 0.21 µm	54.85 ± 2.33 µm
Bismuth Oxide 120 mg FC Tabs	14.35 ± 0.07 µm	27.00 ± 1.41 µm	43.25 ± 0.35 µm
RSD%	0.25	14.46	16.72

## Data Availability

Not applicable.
